# How do general practitioners contribute to preventing long-term work disability of their patients suffering from depressive disorders? A qualitative study

**DOI:** 10.1186/s12875-016-0459-2

**Published:** 2016-06-07

**Authors:** Chantal Sylvain, Marie-José Durand, Pascale Maillette, Lise Lamothe

**Affiliations:** School of Rehabilitation, Université de Sherbrooke, Sherbrooke, Canada; Centre for Action in Work Disability Prevention and Rehabilitation, Longueuil Campus, Université de Sherbrooke, 150 Place Charles LeMoyne, Longueuil, QC J4K 0A8 Canada; Department of Health Administration, School of Public Health, Université de Montréal, Montréal, Canada; Public Health Research Institute of Université de Montreal, C.P. 6128, Succursale Centre-ville, Montreal, QC H3C 3J7 Canada

**Keywords:** Family practice, General practice, Primary care, Depression, Long-term work disability, Return to work, Sick leave, Sickness certification, Doctor-patient relationship, Mental health

## Abstract

**Background:**

Depression is a major cause of work absenteeism that general practitioners (GPs) face directly since they are responsible for sickness certification and for supervising the return to work (RTW). These activities give GPs a key role in preventing long-term work disability, yet their practices in this regard remain poorly documented. The objectives of this study were therefore to describe GPs’ practices with people experiencing work disability due to depressive disorders and explore how GPs’ work context may impact on their practices.

**Methods:**

We conducted semi-structured individual interviews with 13 GPs and six mental healthcare professionals in two sub-regions of Quebec. The sub-regions differed in terms of availability of specialized resources offering public mental health services. Data were anonymized and transcribed verbatim. Thematic analysis was performed to identify patterns in the GPs’ practices and highlight impacting factors in their work context.

**Results:**

Our results identified a set of practices common to all the GPs and other practices that differentiated them. Two profiles were defined on the basis of the various practices documented. The first is characterized by the integration of the RTW goal into the treatment goal right from sickness certification and by interventions that include the workplace, albeit indirectly. The second is characterized by a lack of early RTW-oriented action and by interventions that include little workplace involvement. Regardless of the practice profile, actions intended to improve collaboration with key stakeholders remain the exception. However, two characteristics of the work context appear to have an impact: the availability of a dedicated mental health nurse and the regular provision of clinical information by psychotherapists. These conditions are rarely present but tend to make a significant difference for the GPs.

**Conclusions:**

Our results highlight the significant role of GPs in the prevention of long-term work disability and their need for support through the organization of mental health services at the primary care level.

**Electronic supplementary material:**

The online version of this article (doi:10.1186/s12875-016-0459-2) contains supplementary material, which is available to authorized users.

## Background

Work is a key part of mental health and of the well-being of individuals and societies [1]. For most people, remunerative employment is a determinant of their self-esteem and social status, and contributes to their material well-being and social participation [2]. However, it is estimated that, in Canada, over 10 % of the labour force suffers from at least one mental health disorder [3], which can affect their work participation [4, 5] and result in significant costs for society. For example, in Switzerland, it is estimated that workday losses and disability insurance account for 55 % of all costs associated with depression [6].

Although the majority of people return to work (RTW) after a short-term absence, recent data from the Netherlands show that 56 % of people with a mood disorder are still absent 6 months after their initial sick leave and 30 % after 1 year [7]. When prolonged absences occur, the costs are not only financial but also human and social, arising from consequences such as isolation and secondary anxiety from apprehension about returning to work and dealing with co-workers’ reactions [8].

General practitioners (GPs) are among the health professionals most often consulted by people suffering from depressive disorders [9–11], ranging from major depressive disorders to subclinical states [12]. In legislation such as that in Canada, GPs are responsible for prescribing sick leave in order for it to be recognized by the employer and for the patient to meet the eligibility requirements of the disability plan [13]. They must then decide on the patient’s functional limitations as well as the timing and conditions of the RTW. Yet practice guidelines for depression provide little guidance for interventions leading to RTW [14–16].

Once sick leave is certified, the GPs have to interact more or less directly with stakeholders in three different systems: compensation, work environment and health care. Concerns may arise due to divergent interests concerning work disability and the RTW [17–19]. For example, insurers are interested in reducing the length of absences to control their costs (e.g., compensation payments) while remaining competitive for their client (the employer). Employers, in turn, need to control their production costs, but also maintain a satisfactory work environment for all workers. Finally, for many healthcare providers, the priority is to restore their patients’ health and, secondarily, to facilitate the RTW [20]. These stakeholders’ divergent interests may result in a lack of concerted action and have a negative impact on patients. A recent metasynthesis showed that a lack of coordination among stakeholders was one of the principal components of the RTW process experienced by people with common mental health disorders and one of the main obstacles the patients mentioned [8].

According to Knauf and Schultz [21], current models of RTW conceptualize disability as a multidimensional concept and a result of the interaction among medical, psychosocial, environmental and ergonomic factors, within a system-based approach. The best practices principles for RTW interventions synthesized by Pomaki et al. [22] for workers with mental health conditions reflect these models. At the disability management level, they identified three principles. The first is the need to ensure RTW coordination and structured, planned and close communication between main stakeholders. The second is the need to apply RTW practices such as practices that activate the worker and help keep the person engaged and focused on RTW. The third is that work accommodations should be seen as an integral part of the RTW process, while recognizing that their effectiveness depends on the implementation context [22]. At the individual level, Pomaki et al. found that facilitating access to evidence-based treatment reduces work absence, especially when interventions are combined with counseling about RTW.

To date, we know very little about how these principles translate into GPs’ practices with patients suffering from depressive disorders. To our knowledge, only Macdonald et al. [23] have specifically targeted the views of GPs in Scotland regarding sickness certification in depression [23]. However, this research focused on the daily challenges faced by GPs rather than thoroughly documenting their practices aimed at preventing long-term work disability. Furthermore, a clear portrayal of GPs’ practices should take into account their work context – especially the availability of resources inside and outside the primary healthcare clinic (PHC) – because of its known impact on their practices. According to Anthony et al. [24], for example, the availability in the GPs’ practice environment of mental health specialists with whom they have developed working relationships is a crucial element in GPs’ decisions to refer their patients and treat them with the ongoing collaboration of the specialist. Regarding the management of chronic care diseases, a review by Nolte and McKee [25] concluded that the on-site availability of other health professionals, especially nurses, was relevant since their presence provides benefits in terms of the integration of care. To our knowledge, the factors in GPs’ work context that they perceive as impacting on their practices with people experiencing work disability due to depressive disorders had never been explored to date.

Our qualitative study therefore sought to describe GPs’ practices with people experiencing work disability due to depressive disorders and to explore how GPs’ work context may impact on these practices. The following sections describe the research methods used and present the results obtained. We then continue with a discussion of our key findings and conclude with recommendations for the future.

## Methods

We used a qualitative approach, which is appropriate when little is known about a research topic that could be illuminated by insight into the experiences and perceptions of the people most closely concerned [26]. We conducted semi-structured individual interviews with GPs as our main source of information and with mental healthcare professionals as a complementary source. The study was carried out in the Montérégie region of Quebec (Canada), which is an urban area located on the south shore of Montreal and characterized by a large variety and quantity of public health resources that are inequitably distributed [27].

We recruited participants from two geographical sub-regions (A and B) that differed significantly in terms of availability (low or high) of specialized resources offering public mental health services. These two sub-regions were selected with the input of the administrators of the 11 sub-regions in Montérégie. The criteria applied to select the two sub-regions were the wait time for a consultation with a psychiatrist and the wait time for an appointment with a psychotherapist, both in public services. In the sub-region selected for its low availability of specialized resources (sub-region A), the wait time was more than six months for a psychiatric consultation with no possibility of a one-on-one intervention with a psychotherapist. In the sub-region selected for its high availability of specialized resources (sub-region B), the wait time was less than three months for a psychiatric consultation and less than one month to be seen by a psychotherapist. In both sub-regions, the inclusion criteria for GPs were (1) having a diversified practice and (2) working in a primary healthcare clinic (PHC) located in one of the two selected sub-regions. No exclusion criteria were applied. Information about the study and ways to participate was transmitted by the two administrators of the selected sub-regions to GPs working in PHCs located in their respective sub-region. GPs interested in participating had to contact the principal investigator to schedule an appointment at a time of their choosing and at their office. By recruiting the GPs in this way, it was expected that those who would come forward would be GPs interested in mental health practices and RTW, and it was precisely these practices we wanted to document. Two reminders were issued to boost participation.

We also recruited mental healthcare professionals working in each of the two sub-regions in order to gain a thorough understanding of how mental health services, including relationships with GPs, were organized there. We began by contacting the person in charge of mental health services in each sub-region. Then, we asked for the names of the healthcare professionals with the most intimate knowledge of both the local public mental health services offered and the procedures for working with GPs in their sub-region. Permission to contact them was also requested. A final addition to the data corpus was various documents (clinical and administrative) regarding the organization of mental health services in each sub-region.

Ethics approval was granted by the committees having jurisdiction over the two selected sub-regions: the Étienne-Lebel Ethics Committee and the CSSS Richelieu-Yamaska Ethics Committee. All participants signed an informed consent form.

Two members of the research team (CS, PM) carried out semi-structured interviews with each participant between December 2013 and August 2014. Neither had had relationships with any of the participants prior to the study. Each interview was audio-recorded with the participant’s consent. The interview guide was composed of open questions and unstructured probes to clarify and expand what was being said by the participants. The GPs were questioned about their role when treating working patients with depressive disorders, on the spectrum from major depressive disorders to sub-clinical states. We asked about their practices when assessing sick leave, during follow-up and when planning RTW. We also asked about their experiences and opinions regarding collaboration with stakeholders (mental healthcare professionals, employers and insurers) and how they interact with them. Finally, we asked about resources they use to support their practices for managing sick-leave patients. During the interviews with the mental healthcare professionals, we asked about their role with working patients suffering from depressive disorders, their experiences interacting with their patients’ GPs and the factors hindering or facilitating this interaction. The two interview guides can be viewed online (Additional file 1).

In accordance with recommendations by Miles et al. [28], data analysis was carried out through an iterative process of data reduction, data display and conclusion drawing. Four steps were performed. The first three steps were carried out after completing a first round of interviews, i.e., nine of the 13 interviews with the GPs. First, these interviews were transcribed verbatim, anonymized, and imported into AtlasTi software. Second, thematic analysis was carried out independently by CS and PM based on codes established using the interview guide. For example, codes were divided into three thematic categories: practices when assessing sick leave, during follow-up and when planning RTW. For each category, we grouped our data according to the type of information collected by GPs, how it is collected, decisions made, which messages are given to patients, what other interventions are carried out, what and how interactions with stakeholders are conducted and with what means. Within each thematic category, we added codes for emerging themes. Divergences between the two coders were discussed until a 70 % level of agreement was reached, and PM coded the following interviews. Third, we used different types of matrices [28] to analyze patterns between GPs’ practices, first by sub-region, then all together. To validate the soundness of our interpretations, each emerging pattern was submitted to a search for contradictory data, and the results of the search were discussed in depth by the expanded team. The next four interviews with GPs were then analyzed, which helped highlight the recurrence in themes, until data saturation was reached. The interviews with mental healthcare professionals were also transcribed verbatim, anonymized and included in our data corpus, along with the various documents collected. Although we did not perform thematic analysis on these data, we referred to them periodically to contextualize and inform our understanding of GPs’ practices.

To ensure scientific rigour during data collection and analysis, we kept track of all decisions we made by writing field notes and memos, thus ensuring process auditability [28]. These notes were reviewed periodically and discussed during team meetings to ensure methodological coherence [29]. We also ensured the credibility of our findings in two ways: first, by gaining knowledge of the GPs’ practice environment through the interviews we conducted with mental healthcare professionals and through the documents we collected; and second, by obtaining comments from GPs during a meeting held in each region, where we presented our preliminary findings to a group of 10 to 15 GPs from among those whom we had interviewed during our study. The fact that they perceived our findings as reflecting their day-to-day practices left us confident about their credibility. Finally, in terms of fittingness, we were very careful to provide a detailed enough description that would allow for evaluation of its applicability to another context.

## Results

As shown in Table 1, we interviewed 13 GPs working in six PHCs: three located in sub-region A (low resources) and three in sub-region B (high resources). All but one of the GPs were women. The interviews ranged from 33 to 72 min in duration, for an average of 53 min. We also interviewed six mental health professionals: three in sub-region A and three in sub-region B. They came from different professions (nurses (*n* = 2), social worker (*n* = 1), psychologist (*n* = 1), psychiatrist (*n* = 1) and physician (*n* = 1). These interviews ranged from 37 to 57 min in length, for an average of 48 min.

**Table 1:** Characteristics of participating GPs and their Primary Healthcare Clinics (PHCs) by sub-region

	Characteristics of participating GPs (*n* = 13)			Characteristics of the PHCs (*n* = 6) where the GPs practice		
	# GPs	Experience as GP (yr.)	Specific interest in mental health	# PHCs	No of GPs	Other healthcare professionals
Sub-region A (low resources)	GP-01	31	yes	PHC-01	6	Nurse
	GP-02	33	no			Psychiatric nurse
	GP-03	34	yes	PHC-02	7	Nurse
	GP-04	18	yes			
	GP-05	4	no			
	GP-06	13	no	PHC-03	12	Nurse
	GP-07	10	yes			
Sub-region B (high resources)	GP-08	4	no	PHC-04	6	-
	GP-09	25	no			
	GP-10	33	no	PHC-05	10	Nurse
	GP-11	18	yes			
	GP-12	30	no	PHC-06	9	Nurse
						Psychiatric nurse
	GP-13	5	no			Nutritionist

We present the results obtained regarding the GPs’ practices in two sections. The first section describes practices shared by all the participating GPs, while the second section focuses on practices that differentiated the GPs into two groups.

### Shared GP practices for managing sick leave

The practices shared by all the interviewed GPs were grouped under three dimensions: assessment of sick-leave relevance, treatment of symptoms and collaboration with stakeholders.

#### Assessment of sick-leave relevance

For all the GPs, the information essential to determining the relevance of a sick leave is the intensity of the symptoms and the magnitude of their functional repercussions. However, none of the GPs (with the exception of one) utilize standardized measurement tools to collect this information, relying instead on the clinical interview to make their judgment. They reassess patients at least every 4 weeks.

#### Treatment of symptoms

Our results showed that regular physical activity and psychotherapy are an integral part of the non-pharmacological treatment recommended during sick leave for depressive disorders. When steering patients toward psychotherapy services, the GPs’ preferences are employers’ resources (Employee Assistance Program ─ EAP) because of their focus on work issues, and private resources, especially for patients with insurance. Thus, only patients not covered by insurance are referred to public mental health resources. The GPs consider this strategy necessary to bypass the often-lengthy wait times for public psychotherapy services, regarded as causing inconveniences to the patient, but also to the GP, as evidenced in the following comments:

*“When the diagnosis is made, if I could send my patient the following week to the psychologist* [public services]*, so treatment starts immediately, it would save time. Whereas, well, it'll be three months later, so it’s three months lost for the employer, for the insurance, and then, the insurer isn’t happy because the leave is extended. We're really stuck.”* (GP from sub-region B)

Interestingly, this reasoning was found to be equally present in the two areas, one offering better access to public resources specialized in mental health and the other where access is more limited.

#### Collaboration with stakeholders

Overall, the GPs’ collaboration with the various stakeholders appears minimal, sometimes even nonexistent. Regarding psychotherapists, the GPs consider it important to receive clinical information from them to ensure consistency between their respective interventions, but such exchanges are very rare and limited to problematic situations.

*“When I feel like we’re not heading for the same goal, then I’ll tell the patient, ‘Listen, I'd like your therapist to send me a message to see where he’s at and where he’s going, because I have the impression that we’re not on the same wavelength.’ It’s in this case that I’ll do it. But when I feel we’re on the same wavelength; they don’t do it spontaneously and I don’t ask most of the time.”* (GP from sub-region A)

Information is never exchanged directly with employers, mostly to preserve confidentiality of information, and is not even seen as relevant. Finally, an exchange of information with the insurer is considered important, but solely to facilitate access to specialized services (such as rehabilitation) that are otherwise difficult to access. In fact, these exchanges are limited to periodically filling out forms, as requested by the insurer.

Table 2 presents a summary of these shared practices.

**Table 2:** Summary of shared GP practices for managing sick leave

Dimensions	Shared practices
*Assessment of Sick-leave Relevance*	
Information essential to supporting the relevance of sick leave	Intensity of the symptoms, magnitude of the functional repercussions
	Rarely evaluated using standardized measurement tools
Frequency of re-assessment	Re-assessment at least every 4 weeks
*Treatment of Symptoms*	
Type of non-pharmacological treatment recommended during sick leave	Psychotherapy
	Regular physical activity
GPs’ order of preference when steering the patient toward psychotherapy services	Employer resources (Employee Assistance Program ─ EAP)
	Private resources
	Public resources
*Collaboration with Stakeholders*	
Exchange of information with psychotherapists	Not frequent but seen as important to ensure consistency between psychotherapist’s and GP’s interventions
	What is seen as most important is receiving clinical information from the psychotherapist
Exchange of information with employers	Never done and not seen as relevant
Exchange of information with insurers	Limited to periodically filling out forms
	Seen as important, but solely to facilitate access to specialized services that are otherwise difficult to access (e.g., rehabilitation)

### Differentiating GP practices for managing sick leave

Based on a set of practices showing variations among the GPs, we identified two practice profiles for managing sick leave. In short, the GPs in profile 1 (*n* = 8) tend to focus on the person interacting with his/her environment, while those in profile 2 (*n* = 5) focus solely on the person. These differences are not related to the geographical area where the GPs work, although we expressly selected two sub-regions that differed significantly in terms of availability of specialized resources offering public mental health services.

The dimensions used to differentiate these two profiles were the following: assessment of the contribution of work stressors to work disability, sense-giving regarding sick leave and RTW, and the planning of steps towards RTW. The following section describes profiles 1 and 2 in terms of these three dimensions.

### Profile 1

#### Assessment of the contribution of work stressors to work disability

The GPs in profile 1 tend to view work stressors as something that exceeds the patient’s current adaptive capacity but that could diminish in the short term. This way of factoring work environment stressors into their decision to authorize sick leave leads them to either postpone the decision or to give only short-term sick leave, which can sometimes cause disagreements with the patient.

*“Yesterday, I had a patient who has a conflict and so she wanted sick leave, but I told her, ‘it won’t help you because, in a month, the conflict won’t be any more resolved,’ so it’s [best] to keep working, and to try to find solutions while continuing to work.”* (GP from sub-region B).

#### Sense-giving regarding sick leave and RTW

In terms of the message given to the patient, in profile 1, the GPs explain sick leave as being part of the treatment and of limited duration. The GPs in this profile said they talk about RTW right from the beginning of the sick leave and present it as part of the treatment and inevitable.

*“And often, right from the first moment, I say, ‘Well, I'll put you on sick leave, but be aware that returning to work is part of the treatment’.”* (GP from sub-region B)

Finally, during patient follow-up, the GPs in profile 1 present RTW as something that will help patients wind up their recovery and encourage them to contact co-workers or the supervisor to reduce apprehensions about the RTW.

*“People can never be cured before they return to work. To me, it’s nonsense to think that anyone can be completely well before returning to work because if one hasn’t faced the work environment, the anxiety and uncertainty will remain.”* (GP from sub-region A)

#### Planning of steps towards RTW

Finally, in terms of how the work environment is taken into account when preparing for the RTW, the GPs in profile 1 ask patients to contact their supervisor to discuss a realistic RTW plan before defining it formally.

*“I tell people, ‘Talk to your employer ahead of time, find out what’s possible’.”* (GP from sub-region B)

In addition, the GPs are proactive in recommending specialized occupational rehabilitation resources if they feel that work stressors may be significant and persistent.

### Profile 2

#### Assessment of the contribution of work stressors to work disability

In profile 2, the GPs tend to see work stressors as having an adverse effect on the patient’s health, which induces them to give sick leave in order to remove the patient from this adverse effect. These GPs reported that disagreements with patients about the decision to recommend time off work are rather rare.

#### Sense-giving Regarding Sick Leave and RTW

In profile 2, the GPs explain sick leave as a time to rest, giving no specific message about RTW at the beginning of sick leave and saying nothing to encourage contact with the workplace during follow-up.

*“Patients are generally very anxious about returning to work so I tend not to address that. It’s certainly on my mind that the sooner the better, but I don’t talk about it with patients.”* (GP from sub-region A)

Furthermore, complete severing of contact with the workplace is seen as preferable when major apprehensions regarding the RTW are present.

[I say to the patient,] “If you tell me that you’re returning to work and that it will increase your stress, it would be better to stay away a bit longer and do something else.” (GP from sub-region A)

#### Planning of steps towards RTW

In terms of how the work environment is taken into account when preparing for the RTW, the GPs in profile 2 said they draft the RTW plan around the employer’s constraints as understood by the patient. They take no particular action proactively to mobilize specialized occupational rehabilitation resources in order to prevent work stressors from becoming significant RTW obstacles.

Table 3 provides a detailed overview of the two GPs’ practice profiles.

**Table 3:** Summary of the two GPs’ practice profiles for managing sick leave

Dimensions	Practice profile 1	Practice profile 2
*Assessment of the Contribution of Work Stressors to Work Disability*		
How work environment stressors are taken into account in a decision to authorize sick leave	Work stressors seen as exceeding the patient’s current adaptive capacity, but as possibly diminishing over the short term	Work stressors seen as having an adverse effect on the patient’s health - Give sick leave to remove the patient from the adverse effect of the stressors
	- Postpone the decision - Give short-term sick leave	
*Sense-giving regarding Sick Leave and RTW*		
Meaning of sick leave as explained to the patient	Sick leave is part of the treatment and of limited duration	Time to rest
Message passed on early about the RTW	RTW presented at beginning of sick leave (i) as part of the treatment and (ii) as inevitable	No specific message
Messages about the RTW passed on during follow-up	RTW helps wind up the recovery	Complete severing of contact with the workplace is preferable
	Need to contact co-workers or the supervisor to reduce apprehensions about RTW	
*Planning of Steps towards RTW*		
How the work environment is taken into account during preparation of the RTW	Patient asked to contact his/her supervisor to discuss a realistic RTW plan	Drafting of the RTW plan, taking into account the employer’s constraints, as understood by the patient
	Specialized occupational rehabilitation resources recommended if the stressors are significant and persistent	
		No particular action to proactively mobilize specialized occupational rehabilitation resources

### Characteristics of the work context impacting on GP practices

As mentioned earlier, it was not possible to discern a pattern linking GPs’ practices to the level of availability of specialized mental health resources in their area. However, we found two contextual characteristics that appear to impact on GPs’ collaboration with the various stakeholders: the availability of a dedicated mental health nurse and the provision of clinical information by psychotherapists. These conditions are rarely present but tend to make a significant difference for the GPs.

#### Availability of a dedicated mental health nurse

We observed this condition in only two PHCs, with one in each sub-region. In these two PHCs, a nurse carried out both liaison work with other healthcare professionals and short-term clinical interventions in close collaboration with the GPs. The importance of this specialized mental health resource was made clear in this quote from a GP met shortly after the nurse’s departure.

*“For me, it was VERY helpful because after I had seen the patients, the nurse was able to do the follow-up for many things, even for the insurance papers (…) after that she could do the drug monitoring (…), then even if we wanted the psychiatrist’s opinion, she presented the case* [to the relevant parties]*. It's been two weeks since she’s been gone and, for me, it's placed a huge burden on my shoulders.”* (GP from sub-region A)

The nurse’s departure turned out to be related to a disagreement among the GPs on how to distribute the available resources. However, it appears that the tasks entrusted to the mental health nurse had not been taken over by the PHC nurse. This nurse focused instead on follow-up for clients with chronic health problems, but not for mental health cases.

#### Provision of clinical information by psychotherapists

This facilitating condition involves systematic and regular transmission of clinical information to the GPs by the psychotherapists. However, this was done only by professionals working in public services in sub-region B.

*“They [public resources stakeholders] certainly are now sending us written reports, conclusions, we can talk if necessary (…). Private psychologists and employee assistance programs don’t write to us.”* (GP from sub-region B)

*“I think the psychologist is sometimes a better judge as to when the patient is ready to return, compared to us [GPs] who see the patient during a consultation when many things are sorted out.”* (GP from sub-region B)

This condition, which was only documented in sub-region B, remains marginal because, as mentioned earlier, the GPs refer their patients least often to public resources, mainly because of their perceived limited availability.

## Discussion

Overall, our results showed that all the GPs who participated in the study were concerned by the RTW of their patients with mental health problems, but also revealed variations in how this concern is reflected in their practices. Two profiles were identified. The first is characterized by the integration of the RTW goal into the treatment goal right from the authorization of the sick leave and by interventions that include the workplace, albeit indirectly. The second is characterized by a lack of early RTW-oriented actions and by interventions that include little workplace involvement. Regardless of the practice profile, our results showed that GPs’ collaboration with different stakeholders remains the exception.

These results are important because people with depressive disorders make up a substantial part of GPs’ clientele. Furthermore, GPs have a central role in potentially crucial decisions regarding the prevention of long-term work disability in this population, especially in jurisdictions where GPs are responsible for sick leave certification and planning RTW conditions, as was the case in this study.

Profile 1, as shown by our results, is particularly telling in this regard. This profile is consistent with the best practices principles for RTW synthesized by Pomaki et al. [22], especially with the need to apply practices that activate the worker and help keep the person engaged and focused on RTW. By showing that such a profile exists in the day-to-day practices of GPs (who are not specialized in work rehabilitation), it becomes evident that GPs can adopt practices incorporating some of the best current knowledge on work disability prevention, despite their well-known time constraints. The description provided here of the concrete operationalization of this knowledge in GPs’ practices fills a knowledge gap in this field. To the best of our knowledge, apart from the study by Macdonald et al. [23], no study has documented GPs’ views on their sick-leave management practices, in relation to depressive disorders. Occupational health guidelines obviously exist for the management of mental disorders, but the study by Joosen et al. [30] showed that only two of the 14 guidelines identified worldwide have GPs as target users. These two guidelines are from the Netherlands, where GPs and occupational physicians have a shared responsibility for sick leave and RTW. However, this does not reflect the situation in Canada (our research context) or elsewhere, such as the UK. Thus, by describing GPs’ practice profiles that are specific to depressive disorders and consistent with some best practices principles regarding the prevention of long-term work disability, our results suggest a realistic and concrete way to translate this knowledge into GPs’ mental health practices.

Several aspects, however, remain unclear. For example, our results indicate that the GPs in profile 1 convey messages mentioning a RTW to their patients soon after they begin sick leave, but these may be nuanced, depending on the severity of the depressive disorder. Other questions also arise with respect to the strategies that should be implemented simultaneously to preserve a working alliance with the patient. Studies such as those by Money et al. [31] and Nilsen et al. [32] have provided good descriptions of the potential for conflict during the negotiation of sick leave and RTW. It is likely that a proactive approach towards work, as described by profile 1, may generate such conflicts. Occasional disagreements with patients, as reported by the GPs in profile 1, offer an instructive example. The practices that should be given preference to maintain a strong working alliance are therefore an important question, but one that lies outside the scope of our study.

Other important issues raised by our results concern the implications of GP’s practice profile 2. For example, if profile 1 is considered consistent with some best practices principles for RTW, does this mean that profile 2 should be avoided? Under certain circumstances, should GPs favour interventions that include little workplace involvement? Several studies in fact show that certain working conditions can have a deleterious effect on workers’ health and that these pathogenic conditions are present in some workplaces. For example, in the survey conducted by Vézina et al. [33] of a representative sample of Quebec workers,12.7 % of the workers reported being exposed to high psychological work demands, low decisional latitude and low social support at work, a combination of factors associated with high psychological distress. The same is true of workplace bullying, whose prevalence is estimated, according to Nielsen et al. [34], at between 2 and 14.3 %, depending on the measurement method used. In summary, while these data indicate that in some organizations, work sometimes contributes to producing negative health effects, it should not obscure the fact that this is not always so. Although our results do not provide a definitive answer as to the value of profile 2 in such circumstances, the large pool of current knowledge indicates that – provided the work is safe and accommodating – the beneficial health effects of work generally outweigh the risks and that they are more important than the harmful effects of a prolonged absence [1 2]. This would in fact suggest that GPs’ usual conduct should align with profile 1, at least when their patients’ work demands do not threaten their safety. Of course, the assumption here is that GPs have the ability and means to distinguish between work situations that threaten their patients’ safety and situations that do not. This raises the question of concerted action, which is the next point of discussion.

Our results show that regardless of the practice profile they adopted, the GPs rarely work in collaboration with stakeholders in their day-to-day practices. Direct contact (e.g., clinical report, telephone, face-to-face contact) with other health professionals remains the exception rather than the rule, and the relevance of these direct contacts for the GPs is limited to receiving information from other professionals and rarely involves defining a shared goal. Contact with insurers is also limited to the strict minimum, while with employers it is nonexistent.

These findings pose concerns from the perspectives both of quality of care and prevention of work disability. From the quality-of-care perspective, sound evidence currently supports the superiority of a shared care approach over usual care for treating depression at the primary care level [35, 36]. According to Kates et al. [37], shared care is delivered by providers working in different fields, disciplines or sectors, but who support each other and work together to provide complementary services. The situation of a GP and a psychologist belonging to different organizations but treating a common patient, as we have documented in our study, fits this description. However, our results suggest that this organizational model for mental health care is difficult to implement, at least in the PHCs where the GPs we met practice. This finding is consistent with those of other reports [38].

Our results also raise concerns from the perspective of the prevention of long-term work disability, given that RTW coordination and structured, planned and close communication between stakeholders optimize RTW [22]. GPs’ involvement in such concerted action is even more crucial in a medico-administrative context, as in Quebec, where the responsibility for deciding the timing and conditions of RTW rests with the GP. One may well question the GP’s ability, when there is no direct or indirect contact with the work environment other than what the patient says, to make a valid judgment about the person’s ability to return to work. This question is all the more relevant given that our results indicate that GPs do not use standardized scales to monitor symptoms or their functional impact, particularly on work.

Several factors may explain the lack of concerted action in the practices of the GPs who participated in our study. Limited consultation time is one of them. While a known limiting factor [39, 40], it alone seems insufficient to entirely explain the current situation. Like other authors [41–43], we too believe that medical practice is directly impacted by organizational and environmental factors. For example, the fact that GPs are paid on a fee-for-service basis may discourage them from spending valuable time on activities for which there is no associated remuneration, such as clinical discussions. This explanation is supported by a recent study showing that Canadian GPs working in fee-for-service settings spend fewer hours on indirect patient care than do those working in non-fee-for-service settings [44]. The impact of this factor may well be accentuated in a context where there is significant pressure to provide direct services to patients, as in our study. Difficulty communicating directly with mental health specialists (e.g., psychiatrists, psychologists) could also explain the lack of GP involvement in concerted action. According to a survey by Fleury et al. [38], these difficulties are common in medical practices in Quebec and are seen by GPs as contributing significantly to increasing their burden. Finally, with regards to interaction with employers, our results suggest that GPs do not engage in such interaction for confidentiality reasons. Although this is not surprising considering that protecting their patients’ personal health information is a central part of GPs’ code of ethics [13, 45], these results raise some concerns about how to reconcile such high ethical standards with close communication between stakeholders in order to optimize RTW.

Two conditions in the GPs’ work context appear to support their participation in concerted action. The first favourable condition is the presence of a nurse performing liaison and short-term follow-up tasks for people with mental health disorders. As suggested by our results, the fact that in our study it was a mental health nurse dedicated to these patients was a key factor. This is consistent with the results of Anthony et al. [24], whose work showed that GPs having a mental health specialist in their practice environment have more options in terms of collaboration and referral. However, our results show this to be a precarious condition because it depends on the willingness of those involved to prioritize mental health interventions and allocate the necessary financial resources, which was not the case in one of the two PHCs where a mental health nurse had been practicing.

The other favourable condition documented in this study is the establishment of procedural rules for the systematic and regular transmission of information to GPs by public-sector psychotherapists. Our results show this to be a considerable advantage in a context such as Quebec’s, where there are no computerized clinical records shared by providers from different organizations, and where shared care at the primary care level is rudimentary, as ascertained by Fleury et al. [38]. However, it remains a far cry from conditions enabling RTW coordination and structured and close communication between main stakeholders, as recommended by Pomaki et al. [22] in their best practices principles for RTW.

Our study is important because it helps advance knowledge in a field that is still very underdeveloped. We documented GP practices regarding patients with depressive disorders at the assessment, follow-up and RTW planning stages. Our results are thus not limited only to practices regarding sick leave certification, but they are specific to a particular population. However, the nature of our sample poses some limitations. The first limitation concerns the small number of participants. Given our small sample size, we cannot rule out the possibility that the addition of more interviews might have revealed the existence of other GPs’ practice profiles or that other important aspects may characterize the profiles we identified. Also, had there been a larger number of participants in each sub-region, differences in the practices related to the sub-region might possibly have been documented. The second limitation pertains to the fact that our study sample consisted of volunteers. For example, GPs already sensitized to the issue of sick-leave management may have been overrepresented in our sample. One may wonder whether this stems from the overrepresentation of women in the sample. If so, it may mean that the study did not reveal practices that are in fact prevalent in the general population of GPs, even if less common in our study sample.

The choice made to collect data from semi-structured interviews may also be a source of other limitations. For example, we cannot rule out a possible gap between what the GPs said and their actual practices. Also, the decision to collect data through individual interviews rather than direct observation may have led to a tendency among participants to generalize their practices and minimize the variations. Yet it also provides strong assurance of the wealth of information obtained and leaves us confident about the credibility of our results.

## Conclusions

To date, very few studies have attempted to document the practices of GPs for managing sick leave in relation to depressive disorders. Our data identified a set of practices common to all the participating GPs, others that differentiated them, and certain conditions in their practice context that facilitated their ability to work collaboratively with some of the main stakeholders. More research needs to be conducted to validate these findings with a larger sample, through a survey, for example. For now, given the exploratory nature of our study and the international differences existing at the medico-administrative level, we would recommend caution before transferring our results to other jurisdictions. Although our results did not differentiate clear patterns between the characteristics of the practice environment and GPs’ practices, these aspects warrant investigation in future studies. Similarly, it would be interesting to complete the picture by consulting all interested stakeholders, including insurers and employers. Given the magnitude of the individual and societal costs associated with long-term work disability due to depressive disorders and the GP’s central role in limiting this type of disability, these research avenues should be given high priority. They would be yet another step towards a better understanding of the dynamics at play and finding solutions that optimize a safe and timely RTW.

## Abbreviations

GP, General Practitioner; PHC, Primary Healthcare Clinic; RTW, Return To Work

## Additional file

Additional file 1: Interview Questions. (DOCX 16 kb)

## References

[1] Waddell G, Burton AK (2006). Is work good for your health and well-being?.

[2] Black C (2008). Working for a healthier tomorrow.

[3] Dewa CS, Corbière M, Durand M-J, Gatchel RJ, Schultz IZ (2012). Challenges related to mental health in the workplace. Handbook of Occupational Health and Wellness.

[4] Adler DA, McLaughlin TJ, Rogers WH (2006). Job Performance Deficits Due to Depression. Am J Psychiatry.

[5] Hirschfeld RMA, Montgomery SA, Keller MB (2000). Social functioning in depression: A review. J Clin Psychiatry.

[6] Tomonaga Y, Haettenschwiler J, Hatzinger M (2013). The Economic Burden of Depression in Switzerland. Pharmacoeconomics.

[7] Roelen CAM, Norder G, Koopmans PC (2012). Employees Sick-Listed with Mental Disorders: Who Returns to Work and When?. J Occup Rehabil.

[8] Andersen MF, Nielsen KM, Brinkmann S (2012). Meta-synthesis of qualitative research on return to work among employees with common mental disorders. Scand J Work Environ Health.

[9] Lesage A, Bernèche F, Bordeleau M (2010). Étude sur la santé mentale et le bien-être des adultes québécois : une synthèse pour soutenir l’action. Enquête sur la santé dans les collectivités canadiennes (cycle 1.2).

[10] Fleury MJ, Grenier G, Bamvita JM (2014). Determinants and patterns of service utilization and recourse to professionals for mental health reasons. BMC Health Serv Res.

[11] Slomp M, Bland R, Patterson S (2009). Three-year physician treated prevalence rate of mental disorders in Alberta. Can J Psychiatry.

[12] American Psychiatric Association. Diagnostic and statistical manual of mental disorders (DSM-5®). Arlington, VA: American Psychiatric Pub; 2013.

[13] Canadian Medical Association (2013). The treating physician’s role in helping patients return to work after an illness or injury (update 2013).

[14] CANMAT (2009). Guidelines for the management of depressive disorder in adults. J Affect Disord.

[15] Fournier L, Roberge P, Brouillet H (2012). Faire face à la dépression au Québec. Protocole de soins à l’intention des intervenants de première ligne.

[16] NICE. Depression in adults. The treatment and management of depression in adults. London, UK: National Institute for Health and Clinical Excellence; 2009.

[17] De Vries G, Koeter MWJ, Nabitz U, et al. Return to work after sick leave due to depression; A conceptual analysis based on perspectives of patients, supervisors and occupational physicians. J Affect Disord. 2012;136(3):1017–26. doi: 10.1016/j.jad.2011.06.035 [published Online First: Epub Date]|. 10.1016/j.jad.2011.06.035 21774988

[18] Hees HL, Nieuwenhuijsen K, Koeter MWJ (2012). Towards a New Definition of Return-to-Work Outcomes in Common Mental Disorders from a Multi-Stakeholder Perspective. PLoS One.

[19] Anema J, Jettinghoff K, Houtman I, et al. Medical Care of Employees Long-Term Sick Listed Due to Mental Health Problems: A Cohort Study to Describe and Compare the Care of the Occupational Physician and the General Practitioner. J Occup Rehabil. 2006;16(1):38–49. doi:10.1007/s10926-005-9001-4 [publishedOnlineFirst:EpubDate]|. 10.1007/s10926-005-9001-4 16691458

[20] Young AE, Wasiak R, Roessler RT (2005). Return-to-work outcomes following work disability: stakeholder motivations, interests and concerns. J Occup Rehabil.

[21] Knauf MT, Schultz IZ, Schultz IZ, Gatchel RJ (2016). Current Conceptual Models of Return to Work. Handbook of Return to Work. From Research to Practice.

[22] Pomaki G, Franche RL, Khushrushahi N (2010). Best Practices for Return-to-work/Stay-at-work Interventions for Workers with Mental Health Conditions.

[23] Macdonald S, Maxwell M, Wilson P (2012). A powerful intervention: general practitioners’; use of sickness certification in depression. BMC Fam Pract.

[24] Anthony JS, Baik SY, Bowers BJ (2010). Conditions That Influence a Primary Care Clinician’s Decision to Refer Patients for Depression Care. Rehabil Nurs.

[25] Nolte E, McKee M, Nolte E, McKee M (2008). Integration and chronic care: a review. Caring for people with chronic conditions. A health system perspective.

[26] Patton MQ (2002). Qualitative Research & Evaluation Methods (3rd edition).

[27] Pineault R, Levesque J-F, Roberge D (2008). Accessibility and continuity of health services: A study on primary healthcare in Quebec. Research report.

[28] Miles MB, Huberman AM, Qualitative SJ, Analysis D (2014). A Methods Sourcebook.

[29] Morse JM, Barrett M, Mayan M (2002). Verification Strategies for Establishing Reliability and Validity in Qualitative Research International. J Qual Meth.

[30] Joosen MCW, Brouwers EPM, Van Beurden KM, et al. An international comparison of occupational health guidelines for the management of mental disorders and stress-related psychological symptoms. Occup Environ Med. 2015;72(5):313–22. doi:10.1136/oemed-2013-101626 [publishedOnlineFirst:EpubDate]|. 10.1136/oemed-2013-101626 25406476

[31] Money A, Hussey L, Thorley K, et al. Work-related sickness absence negotiations: GPs' qualitative perspectives. Br J Gen Pract. 2010;60(579):721-8. doi:10.3399/bjgp10X532350. 10.3399/bjgp10X532350 PMC2944931 20883621

[32] Nilsen S, Malterud K, Werner EL (2015). GPs’ negotiation strategies regarding sick leave for subjective health complaints. Scand J Prim Health Care.

[33] Vézina M, Cloutier E, Stock S (2011). Enquête québécoise sur des conditions de travail, d’emploi, et de santé et de sécurité du travail (EQCOTESST).

[34] Nielsen MB, Skogstad A, Matthiesen SB (2009). Prevalence of workplace bullying in Norway: Comparisons across time and estimation methods. Eur J Work Organization Psychol.

[35] Gilbody S, Bower P, Fletcher J (2006). Collaborative care for depression: a cumulative meta-analysis and review of longer-term outcomes. Arch Intern Med.

[36] Katon WJ, Seelig M (2008). Population-Based Care of Depression: Team Care Approaches to Improving Outcomes. J Occup Environ Med.

[37] Kates N, Mazowita G, Lemire F (2010). The evolution of Collaborative Mental Health Care in Canada: a Shared Vision for the Future. Can J Psychiatr.

[38] Fleury M-J, Imboua A, Aube D (2012). General practitioners’ management of mental disorders: A rewarding practice with considerable obstacles. BMC Fam Pract.

[39] Letrilliart L, Barrau A (2012). Difficulties with the sickness certification process in general practice and possible solutions: A systematic review. Eur J Gen Pract.

[40] Wynne-Jones G, Mallen C, Main C (2010). What do GPs feel about sickness certification? A systematic search and narrative review. Scand J Prim Health Care.

[41] Geneau R, Lehoux P, Pineault R (2008). Understanding the work of general practitioners: a social science perspective on the context of medical decision making in primary care. BMC Fam Pract.

[42] Beaulieu MD, Haggerty J, Tousignant P (2013). Characteristics of primary care practices associated with high quality of care. Can Med Assoc J.

[43] Borgès Da Silva R, Contandriopoulos A-P, Pineault R (2014). Effects of practice setting on GPs’ provision of care. Can Fam Physician.

[44] Sarma S, Devlin R, Belhadji B (2010). Does the way physicians are paid influence the way they practice? The case of Canadian family physicians’ work activity. Health Policy.

[45] Canadian Medical Association (2004). CMA Code of Ethics.

