# Supplementary material for: How do general practitioners contribute to preventing long-term work disability of their patients suffering from depressive disorders? A qualitative study
Source: BMC Fam Pract. 2016 Jun 7;17:71. doi: 10.1186/s12875-016-0459-2 (PMC4897943; doi:10.1186/s12875-016-0459-2)
Supplement: Additional file 1: — Interview Questions. (DOCX 16 kb) [file 12875_2016_459_MOESM1_ESM.docx]

**Additional file 1: Interview Questions**

**GPs**

*Part 1: Description of Medical Practices in relation to Work Disability, Practice Context and Impacting Factors*

1. Generally speaking, how do you see your role with patients who suffer from a depressive disorder and have difficulty working?
2. Can you describe to me a recent case in which you wondered about the relevance of certifying sick leave for a patient suffering from a depressive disorder?
   1. What factors did you base your assessment on?
   2. What factors posed problems?
   3. How does this case compare with your usual cases?
3. What are your usual practices when you certify sick leave for a patient with a depressive disorder?
   1. What type of treatment do you usually recommend?
   2. What message do you consider important to pass on to these patients?
   3. How often do you re-assess these patients?
   4. What means do you use as a basis for making your decision?
   5. To what degree does the industry sector in which the patient works influence your decision?
   6. What problems do you usually encounter regarding treatment and periodic re-assessments?
   7. What circumstances make your job easier?
4. How do you usually go about managing your patients’ return to work?
   1. At what point do you raise the question of the RTW with your patients? How do you do it?
   2. What problems do you usually run into?
   3. What circumstances make your job easier at this stage?

*Part 2: Experience and Opinion Regarding Concerted Action*

1. In your usual practices, do you have the habit of contacting the employer, insurer, health office or other healthcare providers involved?
2. Do you usually regard these stakeholders as sharing your view of things?
3. Ideally, how should things work?
4. What means are currently lacking but would make it possible to move closer to achieving this ideal?

*Part 3: Conclusion*

1. Of all the possible improvements that we have discussed during the interview, which do you see as priorities in the short term?
2. Is there any other information that you regard as important and would like to add?

**Mental Healthcare Professionals**

*Part 1: General Practice Context*

1. Can you tell me about the functions you carry out here, and how long have you been carrying them out?
2. With which professionals do you usually work?
3. In what percentage of cases does the question of work arise?

*Part 2: Description of Medical Practices in relation to Work Disability and Interaction with GPs*

1. Generally speaking, how do you see your role with the patients who consult you and have difficulty working?
2. Under what circumstances do you tend to interact with the family doctor of these individuals? What has been your interaction experience with them?
3. Are there circumstances that make things easier from your point of view? And circumstances that hinder things?
4. Ideally, how should things transpire?

*Part 3: Conclusion*

1. Of all the possible improvements we have discussed during the interview, which do you see as priorities in the short term?
2. Is there any other information that you regard as important and would like to add?
